# ^68^Ga-DOTA-NT-20.3 Neurotensin Receptor 1 PET Imaging as a Surrogate for Neuroendocrine Differentiation of Prostate Cancer

**DOI:** 10.2967/jnumed.121.263132

**Published:** 2022-09

**Authors:** Wenyu Wu, Fei Yu, Pengjun Zhang, Ting Bu, Jingjing Fu, Shuyue Ai, Qinqin You, Liang Shi, Guoqiang Shao, Feng Wang, Marina Hodolic, Hongqian Guo

**Affiliations:** 1 Department of Nuclear Medicine, Nanjing First Hospital, Nanjing Medical University, Nanjing, China;; 2 Nuclear Medicine Research Department, IASON, Graz, Austria;; 3 Department of Nuclear Medicine, Faculty of Medicine and Dentistry, Palacký University Olomouc, Olomouc, Czech Republic; and; 4 Department of Urology, Drum Tower Hospital, Medical School of Nanjing University, Nanjing University, Nanjing, China

**Keywords:** ^68^Ga-DOTA-NT-20.3, neurotensin receptor subtype 1, prostate cancer, neuroendocrine differentiation, PET

## Abstract

Prostate-specific membrane antigen (PSMA)–negative neuroendocrine prostate cancer (PCa) is a subtype of PCa likely to be lethal, with limited clinical diagnostic and therapeutic options. High expression of neurotensin receptor subtype 1 (NTR1) is associated with neuroendocrine differentiation of PCa, which makes NTR1 a potential target for neuroendocrine PCa. In this study, the NTR1-targeted tracer ^68^Ga-DOTA-NT-20.3 was synthesized, and its affinity to androgen-dependent (LNCap) and androgen-independent (PC3) xenografts was determined. **Methods:** ^68^Ga-DOTA-NT-20.3 was labeled using an automated synthesizer module, and its stability, labeling yield, and radiochemical purity were analyzed by radio–high-performance liquid chromatography. Receptor binding affinity was evaluated in NTR1-positive PC3 cells by a competitive binding assay. The biodistribution of ^68^Ga-DOTA-NT-20.3 in vivo was evaluated in PC3 and LNCap xenografts by small-animal PET imaging. NTR1 expression was identified by immunohistochemistry and immunofluorescence evaluation. **Results:** ^68^Ga-DOTA-NT-20.3 was synthesized successfully, with a yield of 88.07% ± 1.26%, radiochemical purity of at least 99%, and favorable stability. The NTR1 affinity (half-maximal inhibitory concentration) for ^68^Ga-DOTA-NT-20.3 was 7.59 ± 0.41 nM. Small-animal PET/CT of PC3 xenograft animals showed high-contrast images with intense tumor uptake, which revealed specific NTR1 expression. The tumors showed significant radioactivity (4.95 ± 0.67 percentage injected dose per gram of tissue [%ID/g]) at 1 h, which fell to 1.95 ± 0.17 %ID/g (*P <* 0.01, *t* = 8.72) after specific blockage by neurotensin. LNCap xenografts had no significant accumulation (0.81 ± 0.06 %ID/g) of ^68^Ga-DOTA-NT-20.3 at 1 h. In contrast, ^68^Ga-PSMA-11 was concentrated mainly in LNCap xenografts (8.60 ± 2.11 %ID/g), with no significant uptake in PC3 tumors (0.53 ± 0.05 %ID/g), consistent with the in vitro immunohistochemistry findings. Biodistribution evaluation showed rapid clearance from the blood and main organs (brain, heart, lung, liver, muscle, and bone), with significantly high tumor-to-liver (4.41 ± 0.73) and tumor-to-muscle (12.34 ± 1.32) ratios at 60 min after injection. **Conclusion:** ^68^Ga-DOTA-NT-20.3 can be efficiently prepared with a high yield and high radiochemical purity. Its favorable biodistribution and prominent NTR1 affinity make ^68^Ga-DOTA-NT-20.3 a potential radiopharmaceutical for the detection of PSMA-negative PCa and identification of neuroendocrine differentiation.

## Visual Abstract

**Figure:**
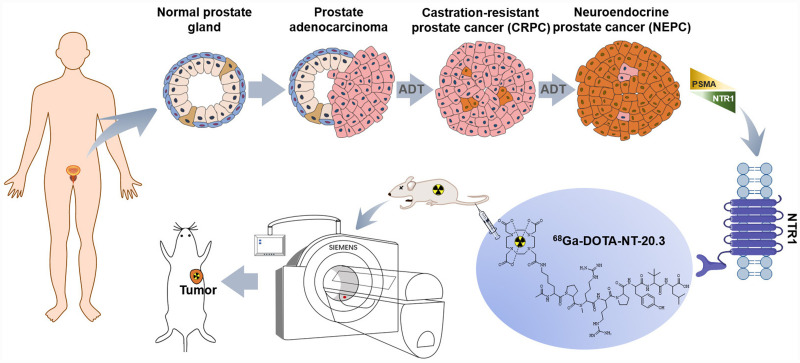

The incidence of prostate cancer (PCa) has increased in line with the aging population and progress in diagnostic modalities (*1*). Patients usually have advanced or metastatic lesions at diagnosis, leading to high mortality. Prostate-specific antigen (PSA) level has been well documented for the diagnosis of PCa and evaluation of tumor response (*2*). However, a PSA increase is largely dependent on the tumor origin; PSA can be increased in benign prostatic hyperplasia and is likely not to increase in poorly differentiated PCa (*3**,**4*). Prostate-specific membrane antigen (PSMA) PET has been widely used clinically and has had merit in the detection of biomedical recurrence, allowing detection of micrometastasis at low PSA values (*5*). PSMA is commonly overexpressed in metastatic castration-resistant PCa (CRPC) and serves as an ideal target for the treatment of PCa (*6*). However, after long-term androgen deprivation therapy, poorly differentiated PCa originating from luminal and basal cells frequently acquires a neuroendocrine phenotype (neuroendocrine PCa [NEPC]), which lacks PSMA expression (*7*). Although the incidence of de novo NEPC is rare (<2%), treatment-driven neuroendocrine differentiation exists in up to 20% of patients with CRPC (*8*). As an aggressive subtype of CRPC, NEPC has a median survival time of less than 1 y because identification is difficult and because the androgen deficiency results in fewer treatment options (*9*). The lethal nature of NEPC is driven by a lack of therapeutic regimens capable of generating durable responses in the setting of extreme tumor heterogeneity at the genetic and cell biologic levels. It is therefore necessary to explore specific molecular targets and efficient therapeutic interventions for the clinical management of NEPC.

The neurotensin/neurotensin receptor (NT/NTR) axis has been identified as an alternative growth pathway in androgen-independent PCa and as a factor in the development of NEPC (*10*). NT, a tridecapeptide released from endocrine cells in the small bowel, stimulates pancreatic and biliary secretion, fatty acid absorption, intestinal motility, and growth of digestive organs (*11*). Additionally, NT secreted from carcinoma cells acts as an autocrine growth factor in response to tumor cell proliferation and migration (*12*). The functions of NT are mediated primarily via 2 G-protein–coupled receptors: NTR subtype 1 (NTR1) (high-affinity receptor) and NTR subtype 2 (low-affinity receptor), whereas NTR subtype 3 serves as a single transmembrane domain localized in the trans-Golgi network (*13*). NTR1 is overexpressed in neuroendocrine differentiation of PCa and may promote neoplastic growth and metastasis after binding with NT produced by neuroendocrine cells in NEPC (*14**,**15*). The latest study showed that NTR1 was expressed in 91.8% of PCa tissues, and all PSMA-negative tissues showed positive NTR1 expression, suggesting the potential complementary role of NTR1-targeted imaging or therapy (*16*). LNCap (androgen-dependent PCa cells) showed negative NTR1 expression, whereas PC3 (androgen-independent PCa cells) had positive expression. Although native NT is sensitive to peptidases, various NT analogs with higher stability have been radiolabeled and used as valuable imaging and internal radioligand therapeutic tools for NTR1-positive tumors (*17*–*20*). Among them, ^68^Ga-DOTA-NT-20.3 is confirmed to be a promising PET imaging probe for NTR1-positive tumors such as pancreatic adenocarcinoma and colon cancer (*21**,**22*). However, ^68^Ga-DOTA-NT-20.3 PET for the quantitation of NTR1 expression in PCa that underwent neuroendocrine differentiation has not been reported. In this study, ^68^Ga-DOTA-NT-20.3 was used to evaluate the neuroendocrine differentiation status in PCa xenografts.

## MATERIALS AND METHODS

### General

Vender-provided information on the chemicals, cells, reagents, and animals, as well as the cell culture and tumor model, is provided in the supplemental materials (available at http://jnm.snmjournals.org). All animal studies were approved by Nanjing First Hospital animal ethical committee and performed according to national regulations.

### Radiolabeling of DOTA-NT-20.3/PSMA-11 with ^68^Ga and Quality Control

An iQS-TS automated module was used for all radiolabeling steps, which were performed as previously described (*21*–*23*) with minor modifications. Briefly, DOTA-NT-20.3 (4.32 nmol, 20 μg) or PSMA-11 (19.72 nmol, 20 μg) dissolved in 1.0 mL of sodium acetate buffer (0.25 M, pH 8.0) and ^68^Ga (370–450 MBq) eluted from the ^68^Ge/^68^Ga generator with 4.0 mL of 0.05 M HCl was introduced into the preheated reactor. The pH of the final labeling solution was 3.5–4.0. After reaction at 95°C for 14 min, the labeled product was concentrated using a disposable Sep-Pak C18 cartridge (Waters), eluted with 0.5 mL of 70% ethanol, and equilibrated with 0.9% sodium chloride injection or fresh medium before use. Quality control of radiopharmaceuticals was performed using radio–high-performance liquid chromatography and radio–thin-layer chromatography (details are in the supplemental materials).

### Determination of Lipophilicity

The shake-flask method was used to determine the partition coefficient of ^68^Ga-DOTA-NT-20.3 in *n*-octanol and phosphate-buffered saline (PBS) (pH 7.4) mixture. The organic and aqueous phases were presaturated 24 h before the experiment, and 500 μL of each layer were added to ^68^Ga-DOTA-NT-20.3 (3.7 MBq) and mixed vigorously for 3 min. The layers were separated by centrifugation at 2,000 rpm (416*g*) for 5 min. Aliquots of 100 μL were removed from each phase and measured in a Wizard γ-counter (PerkinElmer). Calculated logD_7.4_ values were expressed as the mean ± SD from 3 experiments.

### Stability In Vitro

For the stability assay, ^68^Ga-DOTA-NT-20.3 was incubated in PBS (pH 7.4) or fresh human serum at 37°C for 15, 30, 60, 120, and 240 min. Plasma protein was precipitated with isovolumic acetonitrile and removed by centrifugation (12,000 rpm, 13,400*g,* 5 min) after incubation. The supernatants were analyzed by radio–high-performance liquid chromatography after filtering through a Cathivex-GV filter (22 μm; Merck).

### Cell Binding Affinity and Uptake Assay

Human prostate adenocarcinoma PC3 cells were seeded into 24-well plates at a density of 1 × 10^5^ cells per well overnight for the receptor-binding affinity and uptake study of ^68^Ga-DOTA-NT-20.3. ^68^Ga-DOTA-NT-20.3 and NT were diluted to 37 kBq/mL and 10^−8^–10^2^ μM, respectively, with fresh medium. For the receptor-binding affinity assay, ^68^Ga-DOTA-NT-20.3 (37 kBq, 500 μL) was added to each well in the presence of varying concentrations (10^−8^–10^2^ μM, 500 μL) of NT. After incubation for 60 min at 37°C, the medium of each well was removed and the cells were washed twice with PBS. The removed medium and washing PBS were collected to represent the amount of free radioligand. The adherent cells were lysed with NaOH (0.1 M, 200 μL) and harvested after being washed twice by PBS. Finally, the cell-bound radioactivity (3 × 10^5^ cells/mL) and the amount of free radioligand were measured in a γ-counter. The half-maximal inhibitory concentration was calculated using GraphPad Prism software. For the cell uptake study, the trial group PC3 cells were incubated with ^68^Ga-DOTA-NT-20.3 (37 kBq, 500 μL) at 37°C for 15, 30, 60, and 120 min. The blocking group cells were saturated with an excess of NT (1 μM, 500 μL) before addition of ^68^Ga-DOTA-NT-20.3 (37 kBq, 500 μL). The radioactivity of adherent cells (3 × 10^5^ cells/mL) was then measured after they were extracted with NaOH (0.1 M, 200 μL) and washed twice with PBS.

### Small-Animal PET/CT Imaging

The whole-body distribution of the tracer in tumor-bearing mice was examined with an Inveon small-animal PET/CT scanner. ^68^Ga-DOTA-NT-20.3, ^68^Ga-PSMA-11, and NT were diluted to 37 MBq/mL, 37 MBq/mL, and 2 mg/mL, respectively, with 0.9% sodium chloride injection. PC3-xenografted mice (18–25 g) were injected with ^68^Ga-DOTA-NT-20.3 (7.4 MBq, 200 μL) via the tail vein under isoflurane anesthesia, and 10-min static PET images were acquired at 0.5, 1, 1.5, 2, and 4 h after injection. For the blocking group, the mice were pretreated with an excess of NT (20 mg/kg of body weight, 200 μL) via the tail vein 15 min before injection of ^68^Ga-DOTA-NT-20.3 (7.4 MBq, 200 μL), and static PET images were acquired at 1 h after injection. PC3- and LNCap-xenografted mice were imaged at 1 h after receiving ^68^Ga-DOTA-NT-20.3 (7.4 MBq, 200 μL) or ^68^Ga-PSMA-11 (7.4 MBq, 200 μL). Images were reconstructed using 3-dimensional ordered-subset expectation maximization with attenuation correction. Regions of interest were drawn over the tumors and main organs, and average signal levels in the regions were measured using an Inveon Research Workplace workstation.

### Ex Vivo Biodistribution

PC3 xenograft mouse models (18–25 g) were used to evaluate the distribution of tracer in blood and major organs. ^68^Ga-DOTA-NT-20.3 was diluted to 37 MBq/mL with 0.9% sodium chloride injection for use. The mice were killed at 5, 15, 30, 60, and 120 min after intravenous injection of ^68^Ga-DOTA-NT-20.3 (3.4 MBq, 100 μL) (*n* = 3 per group). Blood and major organs were harvested immediately, weighed, and counted using a γ-counter. The radioactivity of each sample was calculated as the percentage of injected dose per gram of tissue (%ID/g) and corrected for radioactive decay.

### Immunohistochemical Staining

NTR1 and PSMA expression was evaluated by immunohistochemistry in LNCap- and PC3-derived prostate tumors. PCa tissues were fixed in 4% paraformaldehyde, paraffin-embedded, and sectioned. The sections were then dewaxed and hydrated with xylene and graded alcohol at room temperature before heat-induced antigen retrieval. Endoperoxidase activity was inactivated by 3% H_2_O_2_, and nonspecific sites were blocked with 3% bovine serum albumin. The sections were incubated overnight with NTR1 antibody (catalog number YT3203, 1:200 dilution; ImmunoWay) at 4°C followed by horseradish peroxidase–labeled goat antirabbit second antibody (catalog number GB23303, 1:200 dilution; Servicebio) and then staining with 3,3-diaminobenzidine and counterstaining with hematoxylin solution for 2 min. The samples were finally dehydrated and mounted with neutral resin, and images were acquired using an optical microscope (with camera attached; Nikon Eclipse E100).

### Histologic Analysis

The tissues were fixed in 4% paraformaldehyde and embedded in paraffin for sectioning. Tumor sections were dewaxed, stained with hematoxylin and eosin, fixed with neutral resin after dehydration, and observed using an optical microscope.

### Immunofluorescent Staining

Cells in 12-well culture plates were fixed with 4% paraformaldehyde and permeabilized with 0.5% Triton X-100 (Union Carbide Chemicals and Plastics) in PBS for 20 min at room temperature. Nonspecific antibodies were blocked with 5% bovine serum albumin in PBS for 30 min at room temperature. NTR1 antibody (1:100 dilution) was added to each well and incubated at 4°C overnight to detect NTR1. The sections were then incubated with CY3-labeled goat antirabbit IgG secondary antibody (catalog number GB21303, 1:300 dilution; Servicebio) followed by antifade medium containing 4,6-diamidino-2-phenylindole and were observed under a fluorescence microscope.

### Statistical Analysis

Quantitative data were described as mean ± SD, and differences between groups were analyzed by Student *t* testing or ANOVA using GraphPad Prism software, version 6.0. A *P* value of less than 0.05 was considered statistically significant.

## RESULTS

### Radiosynthesis of ^68^Ga-DOTA-NT-20.3 and ^68^Ga-PSMA-11

**FIGURE 1.**
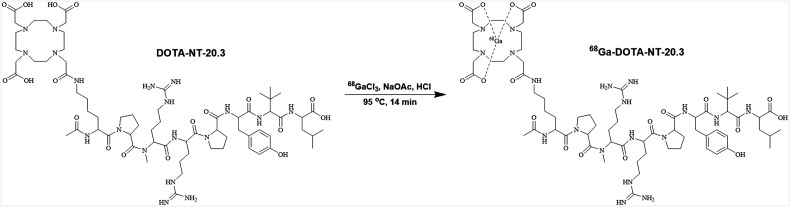
Radiosynthesis and structure of ^68^Ga-DOTA-NT-20.3.

^68^Ga-DOTA-NT-20.3 (Fig. 1) and ^68^Ga-PSMA-11 were labeled successfully within 14 min, with yield rates of 88.07% ± 1.26% and 86.82% ± 2.57%, respectively. The final molar activity (radioactivity of product divided by amount of peptide used) of ^68^Ga-DOTA-NT-20.3 was at least 54.4 GBq/μmol. Radio–high-performance liquid chromatography showed more than 99% radiochemical purity for ^68^Ga-DOTA-NT-20.3 and ^68^Ga-PSMA-11, and the elution times were 13.49 and 8.19 min, respectively (Supplemental Figs. 1 and 2). Radio–thin-layer chromatography of ^68^Ga-DOTA-NT-20.3 showed only 1 spot, with a retention factor of 0.60 (Supplemental Fig. 3).

### Lipophilicity and In Vitro Stability

The lipophilicity of the peptide radiotracer was represented by the partition coefficient of ^68^Ga-DOTA-NT-20.3, determined as a logD_7.4_ value of −3.68 ± 0.14 in *n*-octanol and PBS. The radiochemical purity of ^68^Ga-DOTA-NT-20.3 was still at least 99% after incubation in PBS and human serum at 37°C for 4 h, indicating that the tracer was sufficiently stable for further in vitro and in vivo studies (Supplemental Fig. 4).

### In Vitro Cell Binding Affinity and Uptake

**FIGURE 2.**
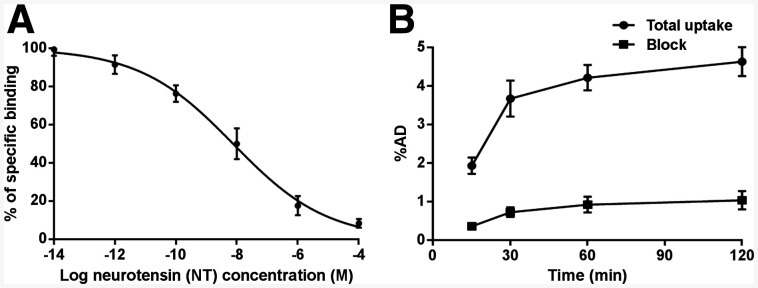
(A) Competitive binding curves for half-maximal inhibitory concentration determination of ^68^Ga-DOTA-NT-20.3 in PC3 cells, using NT as competitive inhibitor. (B) Uptake of ^68^Ga-DOTA-NT-20.3 in PC3 cells. %AD = percentage administered dose.

Competitive cell binding assays revealed that NT inhibited the binding of ^68^Ga-DOTA-NT-20.3 to NTR1-positive PC3 cells in a concentration-dependent manner (Fig. 2A). The half-maximal inhibitory concentration for ^68^Ga-DOTA-NT-20.3 was 7.59 ± 0.41 nM. Cell blocking studies were conducted to evaluate the specificity of ^68^Ga-DOTA-NT-20.3 in vitro (Fig. 2B). The uptake rate of ^68^Ga-DOTA-NT-20.3 by PC3 cells plateaued (4.21% ± 0.33% administered dose) at 1 h of incubation and decreased significantly when blocked with an excess of NT (0.92% ± 0.20% administered dose, *P <* 0.01, *t* = 14.71).

### Small-Animal PET/CT Imaging

**FIGURE 3.**
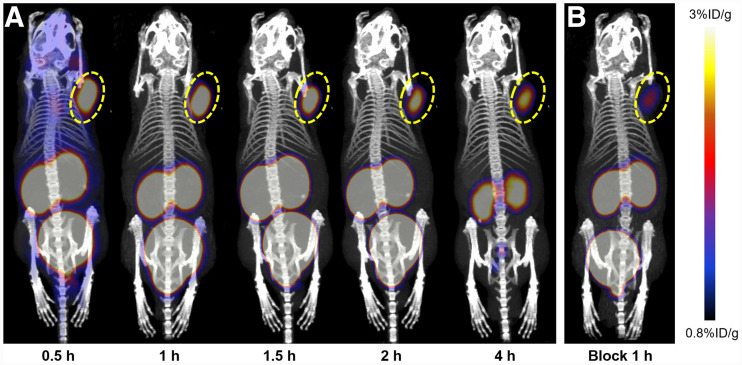
(A) Small-animal PET/CT images of PC3 tumor–bearing mice at different times after injection of ^68^Ga-DOTA-NT-20.3. (B) Blocked by excess NT at 1 h after injection of ^68^Ga-DOTA-NT-20.3, blocking ratio [(total radioactivity uptake − blocked radioactivity uptake)/total radioactivity uptake] was 60.03% ± 6.48%. Tumor site is encircled.

**FIGURE 4.**
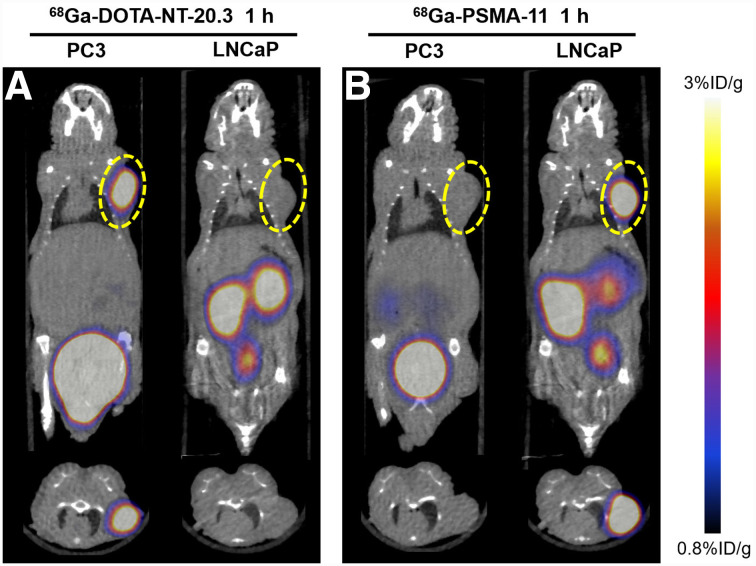
Small-animal PET/CT images of PC3 and LNCap tumor–bearing mice at 1 h after injection of ^68^Ga-DOTA-NT-20.3 (A) or ^68^Ga-PSMA-11 (B). Tumor site is encircled.

PC3-xenograft tumors were clearly visible as early as 0.5 h after injection, and region-of-interest analysis showed tumor uptake of 4.53 ± 1.26 %ID/g (Fig. 3). The tumor-to-background ratio (5.61 ± 0.69) and tumor uptake (4.95 ± 0.67 %ID/g) at 1 h after injection were significantly higher than for the blocking group (1.95 ± 0.17 %ID/g, *P* < 0.01, *t* = 8.72), demonstrating the specificity of ^68^Ga-DOTA-NT-20.3 for NTR1-positive tumors. Quantitative analysis showed that radioactivity peaked in main organs such as heart, lung, brain, bone, and muscle early and then cleared over 1 h. Liver showed no striking radioactivity compared with kidney and bladder, confirming that the tracer was rapidly excreted via the urinary system. LNCap tumor–bearing mice were used as a negative control, and PET imaging demonstrated minimal tumor accumulation of ^68^Ga-DOTA-NT-20.3 (0.81 ± 0.06 %ID/g) (Fig. 4). ^68^Ga-PSMA-11 was subsequently injected into PC3- and LNCap-xenograft mice, and high radioactivity uptake (8.60 ± 2.11 %ID/g) was detected in LNCap but not in PC3 tumors (0.53 ± 0.05 %ID/g). The results indicated that ^68^Ga-DOTA-NT-20.3 specifically targeted NTR1 and could be a promising new tool to complement PSMA PET for the diagnosis of PCa.

### Biodistribution

The metabolic characteristics and targeting specificity of ^68^Ga-DOTA-NT-20.3 in vivo were further evaluated by biodistribution experiments in PC3 tumor models (Table 1). The highest tumor uptake (6.26 ± 0.41 %ID/g) was measured at 60 min after injection and decreased slightly to 3.74 ± 0.56 %ID/g by 120 min. The radiotracer cleared quickly from blood and major organs (brain, heart, lung, liver, muscle, and bone), with significantly high tumor-to-liver (4.41 ± 0.73) and tumor-to-muscle (12.34 ± 1.32) ratios at 60 min. As a consequence of renal excretion, kidney uptake at 30, 60, and 120 min after injection was 23.06 ± 1.94, 24.55 ± 0.98, and 26.08 ± 0.79 %ID/g, respectively, further supporting renal clearance as the primary metabolic pathway of ^68^Ga-DOTA-NT-20.3.

**TABLE 1.** Biodistribution of ^68^Ga-DOTA-NT-20.3 in PC3 Tumor–Bearing Mice at Various Times After Injection

Site	5 min	15 min	30 min	60 min	120 min
Blood	8.11 ± 1.54	4.15 ± 0.59	2.56 ± 0.23	1.12 ± 0.22	0.43 ± 0.08
Heart	7.70 ± 0.90	3.88 ± 0.18	2.78 ± 0.36	1.57 ± 0.18	0.58 ± 0.11
Liver	5.46 ± 1.01	3.17 ± 0.45	2.02 ± 0.48	1.44 ± 0.20	0.86 ± 0.49
Spleen	3.97 ± 0.39	2.81 ± 0.47	1.85 ± 0.43	1.06 ± 0.27	0.41 ± 0.04
Lung	5.18 ± 0.16	3.28 ± 0.62	1.91 ± 0.15	1.20 ± 0.23	0.75 ± 0.15
Kidney	18.36 ± 1.27	21.22 ± 1.95	23.06 ± 1.94	24.55 ± 0.98	26.08 ± 0.79
Stomach	3.56 ± 0.31	2.49 ± 0.35	1.75 ± 0.38	0.91 ± 0.18	0.37 ± 0.05
Intestine	3.28 ± 0.30	2.31 ± 0.92	1.47 ± 0.26	0.77 ± 0.24	0.38 ± 0.04
Pancreas	2.88 ± 0.61	2.03 ± 0.26	1.59 ± 0.60	1.05 ± 0.07	0.36 ± 0.04
Muscle	1.93 ± 0.65	1.44 ± 0.17	0.96 ± 0.08	0.51 ± 0.02	0.32 ± 0.04
Bone	2.27 ± 0.29	2.35 ± 0.52	1.27 ± 0.08	0.86 ± 0.17	0.53 ± 0.17
Brain	1.91 ± 0.39	1.35 ± 0.15	0.88 ± 0.10	0.60 ± 0.05	0.33 ± 0.10
Fat	1.42 ± 0.23	1.11 ± 0.23	0.62 ± 0.11	0.32 ± 0.03	0.32 ± 0.07
Testis	1.62 ± 0.11	1.31 ± 0.05	0.80 ± 0.06	0.42 ± 0.04	0.31 ± 0.10
Tumor	2.81 ± 0.39	3.93 ± 0.43	5.70 ± 0.80	6.26 ± 0.41	3.74 ± 0.56

Data are mean %ID/g ± SD (*n* = 3).

### Immunohistochemical, Immunofluorescent, and Histologic Analyses

**FIGURE 5.**
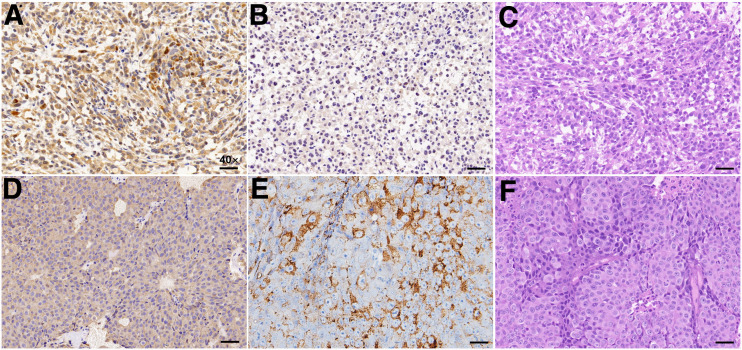
Representative immunohistochemical and histologic images. (A) NTR1 immunohistochemical staining of PC3 tumors. (B) PSMA immunohistochemical staining of PC3 tumors. (C) Hematoxylin and eosin staining of PC3 tumors. (D) NTR1 immunohistochemical staining of LNCap tumors. (E) PSMA immunohistochemical staining of LNCap tumors. (F) Hematoxylin and eosin staining of LNCap tumors. (Scale bar, 10 μm; ×40.)

**FIGURE 6.**
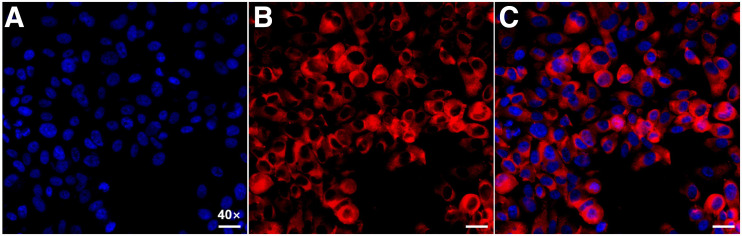
Representative immunofluorescence images. PC3 cells stained with 4-6-diamidino-2-phenylindole showed DNA content (blue) (A) and incubated with fluorescent NTR1 antibody showed NTR1 expression (red) (B). (C) Digital combination (merge) of the 2 previous images is shown to visualize the localization of NTR1 in PC3 cell. (Scale bar, 10 μm; ×40.)

To further validate the NTR1 and PSMA expression in different types of PCa, immunohistochemistry was performed for tumor tissues. PC3 xenografts showed high NTR1 expression levels but no obvious PSMA expression (Figs. 5A and 5B), whereas LNCap tumors showed overexpression of PSMA rather than NTR1 (Figs. 5D and 5E). Tumor immunohistochemistry findings corresponded to the small-animal PET/CT imaging results. Hematoxylin and eosin staining (Figs. 5C and 5F) revealed the different morphologic features of PCa, having irregularly arranged tumor cells that varied in size, with deep staining, obvious atypia, and high mitotic rates. Strong red fluorescence was seen in PC3 cells, confirming the abundant NTR1 expression (Fig. 6).

## DISCUSSION

Despite great progress in the clinical management of localized PCa, metastatic PCa treated with androgen deprivation therapy inevitably develops resistance, leading to CRPC (*24*). Novel antiandrogens (enzalutamide or abiraterone) further prevent tumor progression by inhibiting the reactivated androgen and androgen receptor signaling in CRPC (*25**,**26*). However, prolonged inhibition of the androgen and androgen receptor pathway converts 15%–20% of CRPC into androgen-independent NEPC, with loss of canonic androgen receptor and PSMA expression, clinically resulting in a rapidly progressive disease course and no significant increase in PSA, thus hindering clinical diagnosis and therapy (*7*). PSMA PET/CT and radioligand therapy had limited value for more aggressive PSMA-negative PCa phenotypes in clinical practice. NT/NTR signaling, recruited as an alternative growth pathway in the absence of androgen receptor, plays a crucial role in the proliferation, migration, and invasion of NEPC cells (*10*). Acute NTR1 expression is associated with neuroendocrine differentiation of PCa, making it a promising biomarker that may compensate for the PSMA negativity of NEPC (*16*). Various radiopharmaceuticals developed to trace NTR1 in vivo may aid the early diagnosis, distant metastasis detection, endoradiotherapy, and mechanistic investigation of NEPC (*17*). For this purpose, we radiolabeled ^68^Ga-DOTA-NT-20.3 as an NTR1-targeted radiotracer and evaluated its imaging ability in 2 PCa xenograft models (PC3 and LNCap).

^68^Ga-DOTA-NT-20.3 was efficiently prepared using an iQS-TS automated module, with high yield and high radiochemical purity. The tracer showed good stability in vitro, with a radiochemical purity of at least 99% at 4 h after incubation in PBS or plasma, providing the basis for further biologic evaluations. The lipophilicity of ^68^Ga-DOTA-NT-20.3 was −3.68 ± 0.14, indicating favorable in vivo radiopharmacokinetics, demonstrated by its predominantly renal elimination with little radioactivity in the liver. We also verified the binding affinity and specificity of ^68^Ga-DOTA-NT-20.3 to NTR1 in PC3 cells, which show high levels of NTR1 expression, and further verified the binding by immunofluorescence. ^68^Ga-DOTA-NT-20.3 showed significant time-dependent radioactivity accumulation in PC3 cells. Its binding ability was effectively blocked by an excess of NT within a low-nanomolar range, verifying the specificity of ^68^Ga-DOTA-NT-20.3 for NTR1 in PC3 cells in vitro. A high target (NTR1)-binding affinity is required for high tumor uptake and retention of the radiopeptide, as the basic premise of molecular imaging in vivo.

The specificity of ^68^Ga-DOTA-NT-20.3 was further confirmed by PET imaging in both NTR1-positive/PSMA-negative PC3 and NTR1-negative/PSMA-positive LNCap tumor xenografts. The results showed high and specific accumulation of ^68^Ga-DOTA-NT-20.3 in PC3 tumor lesions at all time points but very low uptake in LNCap-derived tumors. The small molecular size of ^68^Ga-DOTA-NT-20.3 and its hydrophilic nature enable fast clearance of radioactivity from the blood and nontarget tissues, resulting in a high tumor-to-muscle ratio of 5.61 ± 0.69 at 1 h after intravenous injection. Blocking successfully reduced the localization of ^68^Ga-DOTA-NT-20.3 within the tumor because of the presence of an excess of cold NT analogs, clearly demonstrating the receptor specificity of this imaging agent. However, the radioactivity uptake could not be completely blocked by NT, with a blocking ratio of 60.03% ± 6.48% (Fig. 3B), possibly because of an insufficient amount of cold NT; further verifications may be needed. In contrast, ^68^Ga-PSMA-11 PET showed no uptake in PC3-derived tumors but high uptake in LNCap-derived tumors. The different levels of radioactivity uptake in 2 different tumor models can be attributed to the different numbers of NTR1 and PSMA binding sites in PC3 and LNCap cells, respectively. Immunohistochemistry further confirmed high NTR1 expression in PC3-derived tumors and, conversely, high PSMA expression in LNCap-derived tumors.

^68^Ga-DOTA-NT-20.3 showed a prolonged tumor retention time of up to 4 h and quicker clearance from blood, heart, lung, liver, muscle, and other organs or tissues, except kidney—as correlated well with the PET imaging findings. ^68^Ga-DOTA-NT-20.3 cleared predominantly via the renal pathway, leading to accumulation in kidney and bladder. The radioactivity in bladder can be excreted through urine, which is conducive to the detection of paravesical and prostatic bed lesions. Regretfully, kidney may become a dose-limiting organ because of the slower clearance of ^68^Ga-DOTA-NT-20.3. The exact mechanism is not clear, but efforts should be taken to reduce the renal retention and potential nephrotoxicity for future internal radioligand therapy. Biodistribution analysis indicated a high tumor-to-muscle ratio (12.34 ± 1.32) at 1 h after injection, identifying ^68^Ga-DOTA-NT-20.3 as a promising PET tracer for imaging NTR1-expressing tumors. However, compared with the high binding affinity in vitro (half-maximal inhibitory concentration, 7.59 ± 0.41 nmol/L), the radiotracer demonstrated moderate PC3 tumor uptake in vivo (6.26 ± 0.41 %ID/g at 1 h), suggesting that many other factors in addition to binding affinity may affect the tumor uptake. Further systematic investigations are therefore needed to improve the absolute tumor uptake.

## CONCLUSION

This study showed that ^68^Ga-DOTA-NT-20.3 has a high affinity to NTR1 and a favorable distribution and kinetics. The high-contrast images of ^68^Ga-DOTA-NT-20.3 in PC3 xenografts with NTR1-avid expression indicated its potential for detecting poorly differentiated or neuroendocrine differentiation of PCa. The high stability and long intratumor retention of ^68^Ga-DOTA-NT-20.3 hold promise for use in peptide-receptor radionuclide therapy of PCa by exchanging ^68^Ga with the therapeutic radionuclide ^177^Lu/^225^Ac. In addition, ^68^Ga-DOTA-NT-20.3 might be an alternative targeted radiopharmaceutical for identifying neuroendocrine differentiation of PCa. Further preclinical studies are warranted to explore the molecular mechanisms of NTR1 in this context.

## DISCLOSURE

This work was supported by the Jiangsu Provincial Key Research and Development Special Fund (BE2017612), the Nanjing Medical and Health International Joint Research and Development Project (201911042), the General Project of Science and Technology Development Fund of Nanjing Medical University (NMUB2019154), the National Natural Science Foundation of China (82003532), and the second-round fund of Nanjing Clinical Medical Center “Nanjing Nuclear Medicine Center.” No other potential conflict of interest relevant to this article was reported.

## Notes

KEY POINTS **QUESTION:** Can ^68^Ga-DOTA-NT-20.3 serve as an NTR1-targeted radiotracer for the detection of neuroendocrine differentiation in PSMA-negative PCa? **PERTINENT FINDINGS:** ^68^Ga-DOTA-NT-20.3 can be an ideal PET tracer, with favorable characteristics. ^68^Ga-DOTA-NT-20.3 is stable in vitro and has high affinity to NTR1. Cellular uptake studies on the PC3 PCa cell line (NTR1-positive) demonstrated that the uptake is specific. High-contrast images were achieved in PC3 tumor xenografts but not in NTR1-negative/PSMA-positive LNCap tumors. **IMPLICATIONS FOR PATIENT CARE:** In the detection of neuroendocrine differentiation in PCa, ^68^Ga-DOTA-NT-20.3 has merits that may contribute to NTR1-based theranostics and provide a novel strategy for the management of NEPC, especially for neuroendocrine differentiation in metastatic CRPC.
